# Health-related quality of life and emotional distress in patients with dizziness: a cross-sectional approach to disentangle their relationship

**DOI:** 10.1186/1472-6963-14-317

**Published:** 2014-07-22

**Authors:** Steffi Weidt, Annette B Bruehl, Dominik Straumann, Stefan CA Hegemann, Gerhard Krautstrunk, Michael Rufer

**Affiliations:** 1 Department of Psychiatry and Psychotherapy, University Hospital Zurich, Culmannstrasse 8, Zurich CH–8091, Switzerland; 2 Department for Psychiatry, Psychotherapy and Psychosomatics, University Hospital of Psychiatry Zurich, Zurich, Switzerland; 3 Department of Neurology, University Hospital Zurich, Zurich, Switzerland; 4 Department of Otolaryngology, Head & Neck Surgery, University Hospital Zurich, Zurich, Switzerland

**Keywords:** Health-related quality of life, Dizziness, Emotional distress, Impairment

## Abstract

**Background:**

Dizziness is frequently encountered in medical practice, often takes a chronic course and can impair the health related quality of life (HRQoL). However results on the extent of this impairment of HRQoL are mixed. Furthermore, the relationship between dizziness and the HRQoL is only partially understood. The role of clinical symptoms of dizziness and psychosocial factors such as emotional distress on this relationship is for the most part unknown.

**Methods:**

The cross-sectional study evaluated the HRQoL in 203 patients suffering from dizziness, using the Medical Outcomes Studies 36-Item Short-Form Health-Survey (SF-36). The results were correlated with the severity of dizziness, using the Dizziness Handicap-Inventory (DHI), with emotional distress, using the Hospital Anxiety and Depression-Scale (HADS) and with further clinical symptoms and psychosocial parameters. In a multivariate hierarchical regression analysis associated variables which explain significant variance of the mental and physical HRQoL (MCS-36, PCS-36) were identified.

**Results:**

Patients suffering from dizziness showed a markedly reduced mental and physical HRQoL. Higher DHI and HADS scores were correlated with lower MCS-36 and PCS-36 scores. Taken together DHI and vertigo characteristics of dizziness explained 38% of the variance of PCS-36. Overall explained variance of PCS-36 was 45%. HADS and living with a significant other explained 66% of the variance of MCS-36 (overall variance explained: 69%).

**Conclusion:**

Both the physical and mental HRQoL are significantly impaired in patients with dizziness. While the impairment in PCS-36 can be explained by clinical symptoms of the dizziness, MCS-36 impairment is largely associated with psychosocial factors. To improve the patient’s overall well-being significantly and permanently doctors have to keep in mind both, the clinical symptoms and the psychosocial factors. Therefore, in addition to the physical examination doctors should integrate a basic psychological examination into the daily routine with dizziness patients.

## Background

Dizziness is one of the most frequent symptoms encountered in medical practice [1,2], with a prevalence of approximately 20-30% [2-4]. Dizziness is more common in women [2,5]. It is associated with other psychological and physical comorbidities [6,7]. Dizziness can refer to vertigo, presyncope, disequilibrium, or to non-specific feelings such as giddiness or foolishness [8]. Its origin can be vestibular, neurological, cardiovascular or psychological [9]. More than half of the patients suffering from dizziness have non-vestibular diagnoses [2]. Furthermore, in about half of the patients of specialized units for oto-neurological disorders, symptoms of dizziness are not fully explained by identifiable medical illnesses but are related to mental disorders such as anxiety [10-12]. Patients who experience dizziness report a variety of symptoms, including nausea, instability, disruptions of normal activity patterns and emotional distress. These symptoms are suggested to handicap the patients significantly [9,13]. Dizziness often takes a chronic course [2,5,14].

The health related quality of life (HRQoL) is a multidimensional concept, which reflects core components of functioning (e.g. physical, psychological/emotional and social functioning) in the context of medical conditions [15]. HRQoL measurements evaluate the impact of medical conditions on subjective well-being [16]. These measurements are suggested as outcome measures in clinical trials and for assessing the burden of clinical conditions by comparing clinical patients with the general population [16]. One measure assessing HRQoL is the Medical Outcomes Studies 36-Item Short-Form Health Survey (SF-36) [17].

Findings on differences between HRQoL of patients suffering from dizziness and the general population are inconsistent. While some studies of patients suffering from dizziness found impaired mental and physical HRQoL as well as impairment of all eight subscales of the SF-36 [18-20], other studies showed no impairment of some of these scales (e.g. physical function, general health and social function) [21,22]. Reasons for this inconsistency could be differences in the studied samples. While some studies included patients with acute objective measurable medical problems [18,22] others included etiologically heterogeneous patients [20,21] and yet others included patients with Meniere’s disease in which dizziness is only one of three major problems (next to hearing loss and tinnitus) [19].

In addition to these inconsistent results, the associations of clinical symptoms (e.g. duration of illness), psychosocial factors (e.g. emotional distress) and HRQoL have rarely been investigated. As there is evidence that not only clinical symptoms but especially psychosocial factors might be significantly associated to the HRQoL and might play a significant role in treatment outcome [23-25], it seems important to disentangle these associations more precisely. In elderly patients frequency of dizziness, but not the duration of the symptoms correlated with the HRQoL; emotional distress and HRQoL correlated negatively [20]. However, the contribution of these factors to the variance of the HRQoL has not been investigated. In Meniere’s disease, dizziness (not hearing loss and tinnitus) was most strongly associated with a low HRQoL [19]. The duration of Meniere’s disease showed no association with HRQoL [19]. As Meniere’s disease is only one condition of many in which dizziness occurs these results can indicate an association between these factors and the HRQoL but generalization of the findings to the etiologically heterogeneous sample of dizziness patients is difficult.

Altogether the associations between clinical symptom variables (e.g. duration and severity of symptoms), psychosocial factors (e.g. emotional conditions) and HRQoL in patients suffering from dizziness remain unclear. However there is growing evidence that not only clinical symptoms but additional psychosocial factors might influence the perceived HRQoL and the treatment outcome. Therefore, the aim of our cross-sectional study was to identify significant associations between possible predictive clinical symptoms and psychosocial factors and the HRQoL in patients suffering from dizziness. Our sample, which was recruited in the Interdisciplinary Centre for Vertigo and Balance Disorders at the University Hospital of Zurich, was not limited to specific diagnoses. Therefore, the sample was etiologically heterogeneous and included patients with vertigo, non-vertigo and mixed (vertigo and non-vertigo) symptoms of dizziness. We hypothesized that the HRQoL (all subscales) of patients suffering from dizziness is significantly lower than in the general population. We expected that HRQoL in dizziness patients is associated with clinical symptoms (e.g. severity and frequency of symptoms). Furthermore, we expected that psychosocial factors (e.g. emotional distress) influence the variances of the mental as well as physical HRQoL significantly.

## Methods

### Participants

**Figure 1:**
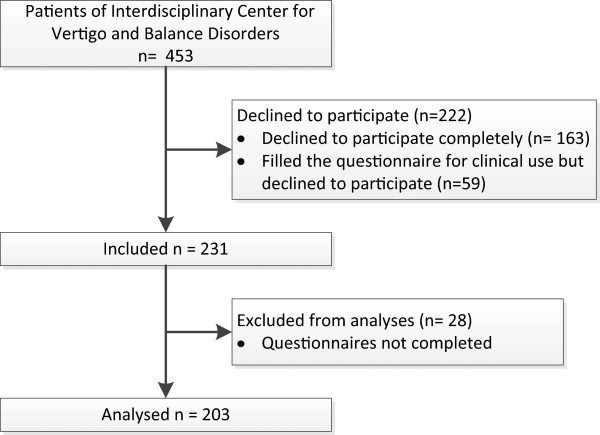
Flowchart of recruitment and retention of participants in the study.

The study was approved by the ethics committee of the Canton of Zurich, and all participants provided written informed consent. Out-patients aged 18–65 years who had been referred to the interdisciplinary Centre for Vertigo and Balance Disorders at the University Hospital Zurich between August 2010 and August 2011 were asked to participate in the study. They were informed, by post of their appointment date and asked to fill out questionnaires to be submitted on the first visit. Four hundred and fifty-three patients were asked to participate. Of these, 163 did not answer the questionnaire and declined to participate (no written consent obtained), 59 did complete the questionnaire for clinical use but declined to participate (no written consent obtained) and 28 partially completed the questionnaire and gave written consent but major parts of the questionnaire were missing (e.g. DHI, SF-36 missing). In total 203 subjects were finally included in the study (Figure 1).

### Measurements

Generic HRQoL was measured using the SF-36 [17]. The SF-36 consists of eight subscales: physical function, role limitation due to physical problems, bodily pain, general health, vitality, social function, role limitation due to emotional problems and mental health. These subscales were merged into two component scores, the physical (PCS-36) and mental (MCS-36) HRQoL. Each component score is standardized (range 0–100), with low scores representing low HRQoL. The German version of the questionnaire is widely used, and normative data for the German general population are available [26]. In most studies, the internal consistency (Cronbach’s alpha) is higher than 0.7 for all subscales and both sum scales [27].

The Dizziness Handicap Inventory (DHI, German version [28,29]) is a disease specific questionnaire for self-assessing the severity of dizziness. It consists of 25 items with a three-step scale (yes = 4, sometimes = 2, no = 0) and total scores ranging from 0 to 100. Three subscales measure the functional (DHIF, 9 items), physical (DHIP, 7 items) and emotional (DHIE, 9 items) handicaps. The German version demonstrates good internal consistency (α = 0.72-0.89) and reliability (test-retest reliability r = 0.92-0.97) and is thus recommended to measure the severity of symptoms in patients suffering from dizziness [18,29].

Emotional distress was assessed with the Hospital Anxiety and Depression scale (HADS, German version) [30,31]. It has fourteen items, which are each rated with 0 to 3 points resulting in a sum score of 0–42 [32]. The scale has been shown to be an effective measure of emotional distress and has acceptable test-retest reliability in patients with vestibular disorders [33,34]. In the light of the latent structure of the HADS, it has been reported to be useful as a general measurement of emotional distress but not to distinguish between anxiety and depression [33].

Clinical (e.g. duration of symptoms) and psychosocial (e.g. working status) characteristics were assessed using a questionnaire developed for clinical use in the Interdisciplinary Centre for Vertigo and Balance Disorder at the University Hospital Zurich. Education ranged from 0 (no secondary school degree) to 3 (university degree). The working status had two dimensions low (0–20 hour worked/week) and full (>20 hours worked/week). Characteristics of dizziness (variable name VNV) were categorized as follows: vertigo, non-vertigo, and mixed (vertigo and non-vertigo characteristics).

### Statistics

Descriptive statistics for different measures were calculated for all participants. SF-36 component scores and subscale scores were compared with population norms [26,27] using *t-*test. The relationship between PCS-36, MCS-36 and DHI sum-score and their relations to clinical symptoms were analysed using Pearson correlation or ANOVA. To deal with the problem of multiple testing, a Bonferroni correction was applied to the results of the correlation analyses. A total of thirty tests were performed, so the threshold for significance was adjusted to 0.05/30 = 0.0017. Hierarchical regression analyses were carried out to test the effects of associated factors on the HRQoL (all included variables of the final model are shown in Table four). The first model for PCS-36 included DHI. In the second model two dummy variables for VNV were added. Models three to eight were built by forcing (method: Enter) the following variables step by step into the model: HADS, sick leave, significant other, working status, pharmacy, and two dummy variables for school. The first model for MCS-36 included HADS instead of DHI. There was no model 2 and the following models were built the same way as for PCS-36 (model 3 DHI instead of HADS). SF-36 component scores (Kolmogorov-Smirnov-test, p ≥ 0.1) as well as all regression residuals were normally distributed (Kolmogorov-Smirnov-test, p > 0.9). Scatterplots of standardized predictive values against standardized residuals of the models indicated no obvious outliers, as well as homoscedasticity and linearity. The Durbin-Watson test for the hierarchical regression (PCS-36: 2.0; MCS-36: 1.7) suggested independent errors as well [35]. The average variance inflation factor was not substantially higher than 1 (PCS-36 and MCS-36 between 1.1 and 1.7), suggesting that the regression was not biased by multicollinearity [36,37]. Furthermore, tolerance of all predictors for PCS-36 was >0.7, and for MCS-36 > 0.6 indicating low multicollinearity as well [38]. The statistical degrees of freedom differed for some parameters (Table 1) due to missing variables. Single missing items were substituted by case mean substitution [39,40]. All calculations were performed by software-package SPSS (version 20).

**Table 1:** Demographic and clinical characteristics of 203 patients with dizziness

	**Mean (median)**	**SD (range)**
**Age, years**	44.6 (45.0)	12.0 (19–65)
**Duration of dizziness, weeks**	174.4 (58)	294.3 (1–1976)
**Frequency of dizziness, times/week**	5.2 (7)	2.3 (0–7)
**Sick leave, days/12 months**	38.2 (2)	92.1 (0–365)
**SF-36**		
MCS-36	41.9	12.2
PCS-36	41.6	10.7
**DHI total score**	46.0	24.4
DHIF	17.1	10.4
DHIP	13.9	7.5
DHIE	15.1	9.3
**HADS**	13.5	8.4
	**Number of Patients**	**%**
**Sex, female/male**	106/97	52.2/47.8
**Working status, yes/no**	140/59	69.0/29.1
**Education**		
No degree/basic school education	36	18.2
Apprenticeship/High school diploma	117	57.6
University degree	45	22.2
**Characteristics of dizziness**		
Permanent	70	34.5
Attacks	125	61.6
vertigo	48	24.2
Non-vertigo	13	6.6
Mixed (vertigo and non-vertigo)	137	67.5

MCS-36: mental health component summary score, PCS-36: physical health component summary score; DHI total: Dizziness Handicap Inventory total sum score, DHIP: DHI physical scale, DHIE: DHI emotional scale, DHIF: DHI functional scale; HADS: Hospital anxiety and depression scale.

## Results

### Characteristics of participants (N = 203)

Two hundred and three patients of the Interdisciplinary Centre for Vertigo and Balance Disorders gave their written consent and completed the questionnaires (Figure 1). These 203 did not differ from the excluded patients (n = 250) in age (*t* = 0.62; mean difference = 0.66, *df* = 451, *p* = 0.54) and sex (chi-square = 2.8, *p* = 0.1). The mean age of the participants was 44.6 (SD 12) years and 106 (52.2%) were female. The mean duration of the dizziness symptoms was 174 (SD 294) weeks. Further socio-demographic and clinical characteristics are given in Table 1.

### Comparison between HRQoL in patients suffering from dizziness with the general-population

**Figure 2:**
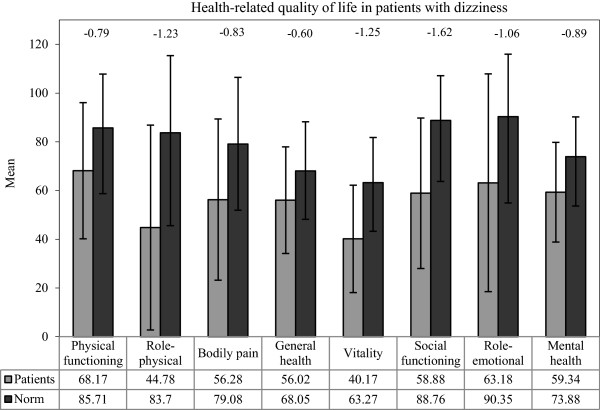
**SF-36 subscale scores of participants with dizziness and the general population.** z-scores are shown at the top of the figure (reference: subjects of the German general population) [26]. All p < 0.001 for patients with dizziness compared with subjects of the German general population. SF-36: Medical Outcomes Studies 36-Item Short-Form Health Survey.

SF-36 component scores were normally distributed. In the current etiologically heterogeneous group of patients suffering from dizziness both sum scales of the SF-36 were significantly lower than normative values of the German population (MCS-36: *t* = −9.0, *df* = 171, *p* < 0.001; PCS-36: *t* = −12.2; *df* = 171, *p* < 0.001). All SF-36 subscales were significantly lower in the patients (all *p* < 0.001). All z-scores of the study participants were 0.6 to 1.6 standard deviations lower than those of the German general-population sample (Figure 2).

### Relationship between HRQoL and dizziness

PCS-36 and MCS-36 correlated significantly with the severity of dizziness (DHI) and emotional distress (HADS, Table 2). These associations were robust, even after Bonferroni correction. The frequency of dizziness (symptoms per week) was moderately correlated with the PCS-36 (however not surviving Bonferroni correction) but not with the MCS-36. The duration of the symptoms was not correlated with the MCS-36 or the PCS-36. Attacks versus permanent dizziness were associated with PCS-36; however not surviving Bonferroni correction. ANOVA showed associations between PCS-36, MCS-36 and vestibular, non-vestibular and mixed characteristics of dizziness (VNV). The association between VNV and PCS-36 was robust after Bonferroni correction. Emotional distress (HADS) and severity of symptoms (DHI) were significantly correlated (Table 2).

**Table 2:** Bivariate correlations between SF-36, DHI, HADS and clinical characteristics of dizziness

	**PCS-36**	**MCS-36**	**DHI**	**HADS**
PCS-36	1			
MCS-36	0.22**	1		
DHI	−0.64***	−0.57***	1	
HADS	−0.32***	−0.82***	0.58***	1
DoD	0.13	−0.09	−0.01	0.004
FoD	−0.20*	−0.03	0.24**	0.04
AvP	0.24**	0.10	−0.25**	−0.07
VNV^a^	F = 7.94***	4.64*	5.65**	5.18**
Age	−0.03	0.01	0.004	−0.02
Gender	0.03	0.13	−0.12	−0.03

Pearson correlations *p < 0.05, **p < 0.01, ***p < 0.001; Bonferroni corrected threshold for significance: p = 0.0017 - correlations surviving Bonferroni correction are bold; ^a^Anvoa for VNV (vertigo, non-vertigo, mixed); SF-36: Medical Outcomes Studies 36-Item Short-Form Health Survey; MCS-36: SF-36 mental health component summary score, PCS-36: SF-36 physical component summary score; DHI: Dizziness Handicap Inventory sum score; HADS: Hospital Anxiety and Depression Scale; DoD: Duration of Dizziness; FoD: Frequency of Dizziness; AvP: attack versus permanent dizziness; VNV: variable for vertigo, non-vertigo and mixed dizziness.

### HRQoL, dizziness and emotional distress

In the hierarchical regression analyses, step by step models were tested to identify factors associated with the PCS-36 and MCS-36 in patients suffering from dizziness (Tables 3 and 4). Only variables which were significantly associated (after Bonferroni correction) with PCS-36 or MCS-36 in the univariate analyses were included in the hierarchical regression models. The first model of PCS-36 included DHI, the second dummy variables for VNV. The first model for MCS-36 included HADS. The step by step hierarchical models for both PCS-36 and MCS-36 (method: Enter) included possible predictive variables in the following order: HADS for PCS-36 and DHI for MCS-36, days of sick leave, significant other, working status, pharmacy, and dummy variables for school (Tables 3 and 4).

**Table 3:** Hierarchical regression model summaries for PCS-36 and MCS-36 as dependent variable

	**PCS-36**				**MCS-36**			
**Models**	**Adjusted R**^**2**^	**R**^**2 **^**change**	**F change**	**Sig F change**	**Adjusted R**^**2**^	**R**^**2 **^**change**	**F change**	**Sig F change**
1	0.35	0.35	69.2	<0.001	0.64	0.65	234.75	<0.001
2	0.38	0.04	4.21	=0.002	-	-	-	-
3	0.39	0.01	2.60	=0.11	0.65	0.01	2.69	0.10
4	0.44	0.06	12.44	=0.001	0.65	0.00	0.08	0.77
5	0.44	0.006	1.28	=0.26	0.67	0.02	9.56	0.002
6	0.44	0.00	0.003	=0.96	0.67	0.00	0.03	0.86
7	0.43	0.003	0.67	=0.42	0.68	0.02	6.45	0.02
8	0.45	0.02	2.83	=0.06	0.69	0.02	4.04	0.02

Model 1 PCS-36: DHI; Model 2 PCS-36: model 1 + dummy VNV variables; Model 1 MCS-36: HADS, Model 3: model 2 + DHI; Model 4: model 3 + sick leave, Model 5: model 4 + significant other; model 6: model 5 + working status, model 7: model 6 + pharmacy, model 8: model 7 + dummy school variables; MCS-36: mental health component summary score, PCS-36: physical health component summary score.

**Table 4:** Model parameters of Model 8 of hierarchical regression for PCS-36 and MCS-36 as dependent variable

**Variables (step at which variable was included PCS-36/MCS-36)**	**Model 8 PCS-36 Beta p(Beta)**	**Model 8 MCS-36 Beta p(Beta)**
DHI (1/3)	**−0.61 (<0.001)**	−0.02 (0.74)
VNV-V (2/-)	**0.22 (0.003)**	/
VNV-NV (2/-)	−0.01 (0.86)	/
HADS (3/1)	**0.24 (0.005)**	**−0.86 (<0.001)**
Sick leave (4/4)	**−0.26 (0.001)**	0.02 (0.73)
Significant other (5/5)	−0.10 (0.14)	**0.19 (<0.001)**
Working status (6/6)	0.01 (0.90)	0.01 (0.81)
Pharmacy (7/7)	0.02 (0.75)	−0.11 (0.06)
School no (8/8)	**−0.19 (0.023)**	**0.15 (0.01)**
School middle (8/8)	−0.05 (0.52)	0.004 (0.95)

MCS-36: mental health component summary score, PCS-36: physical health component summary score, DHI total: Dizziness Handicap Inventory sum score; VNV-V: dichotomous dummy variable for vertigo (1) and non-vertigo or mixed dizziness (0); VNV-NV: dichotomous dummy variable for non-vertigo (1) and vertigo or mixed dizziness (0); HADS: Hospital anxiety and depression score sum score, Sick leave: number of days of sick leave in the last 365 days; Significant other: dichotomous variable for living with an significant other (1) or not (0); Working status: dichotomous variable for working yes (1) and no (2); Pharmacy: dichotomous variable for intake of psychopharmacological drugs no (0) and yes (1); School no: dichotomous dummy variable for no school degree (1) any school degree (0) School middle: dichotomous dummy variable for Apprenticeship or High school diploma (1) and no school degree or higher school degree (0); significant associations are bold.

Psychopharmacological treatment, working status, and middle school (apprenticeship or high school diploma) were not significantly associated with MCS-36 and PCS-36.

Forty-five percent of the total variance of PCS-36 was explained by the included variables. Higher DHI was associated with lower PCS-36 and explained 35 percent of the variance. Vertigo characteristics of dizziness were associated with lower PCS-36 and explained about four percent of the PCS-36 variance. Additional five percent were explained by days of sick leave. Having a higher school degree was significantly associated with higher PCS-36 but the explained variance of PCS-36 was less than one percent. Non-vertigo and mixed (vertigo and non-vertigo) characteristics of dizziness and living with a significant other were not associated with PCS-36.

HADS seemed to show a significant association with PCS-36. But the change of sign between univariate calculations (negative) and the hierarchical model (positive) indicated a suppressor effect. To identify which variable had to be there for the suppressor effect the model was simplified. All non-contributing variables were excluded one by one with no effect on the suppressor effect. The contributing variables were then tested one by one. DHI was identified as the variable that had to be there for the suppressor effect. While HADS had a weak negative correlation with PCS-36 (r = −0.3), once DHI was taken into account, higher HADS scores predicted higher PCS-36 scores. As the univariate correlation was low the suppressor (HADS) rather improved the prediction than contributed to the variance on its own (less than one percent).

Sixty-nine percent of the total variance of MCS-36 was explained by the included variables. HADS explained 64 of that 69 percent, with higher HADS predicting lower MCS-36. Additional two percent were explained by living with a significant other and about 1 percent was explained having no school degree. All other variables showed no significant association with MCS-36.

## Discussion

The current study aimed at differentiating and determining the association between clinical characteristics, psychosocial factors, and the HRQoL in patients with dizziness. In this etiologically heterogeneous group of patients suffering from dizziness, HRQoL was markedly impaired compared to the general population. This impairment was associated with symptom severity (DHI), vertigo characteristics of dizziness, days of sick leave, education (PCS-36) and with emotional distress (HADS), living with someone and education (MCS-36).

The HRQoL of patients suffering from dizziness was markedly impaired in both component scores and all subscales of the SF-36 compared to the general population [27]. This general population is an image of the population aged 18 and above and includes healthy subjects and people with acute and/or chronic diseases. The mean age of our sample (44.7 years) and the norm population (47.7 years) was comparable and the sex-ratio differed only slightly (our sample: 52.2% women, norm population: 55.6% women). While three other dizziness studies [18-20], found similar results, two studies [21,22] found less impairment. One of these two studies [22] included a selected group of elderly patients who had an acute medical condition (Benign Paroxysmal Positional Vertigo). This acute developed medical condition might have caused lower degrees of impairment than the mainly long lasting dizziness (mean 174 weeks) in our sample. Overall, dizziness was related to the physical and mental HRQoL. This impairment seems to be substantial and others as well as our results suggest that the impact of dizziness on HRQoL may be significantly underestimated [9,41].

In the hierarchical regression, 69 percent of the variance of MCS-36 was explained. The most significant associated factor was emotional distress, explaining 64 percent of the variance. Patients suffering from dizziness are often fearful of subsequent dizziness-attacks and the consequences of these attacks [42]. In an attempt to avoid situations that could possibly provoke dizziness, sufferers may restrict their daily activities, and in this way might increase their suffering (lower mental HRQoL) and emotional distress even more. This circle between dizziness, HRQoL, and emotional distress seems to be supported by the current results, which showed that higher emotional distress and lower MCS-36 were significantly associated and that emotional distress in patients with dizziness contributed about 90 percent to the explained variance of MCS-36. A second important contribution variable of MCS-36 was living with a significant other. Living with a significant other was associated with a better MCS-36 and added two percent to its explained variance. The modulating effect of living with a significant other implies that assistance in every-day life by significant others might play an important role in the self-management of chronic diseases. In our study neither gender nor age was associated with MCS-36. Even though there is evidence that dizziness is more common in women and occurs more often in older patients [43] there is limited evidence that this is reflected in the HRQoL [9]. While studies examining heterogeneous groups of patients do not tend to find gender differences regarding generic HRQoL [21] one other study examining patients with Meniere’s disease reported differences between women and men in MCS-36 [19]. However another study found no gender differences in generic HRQoL (SF-12) in Meniere’s patients [44]. Overall, results suggest no significant relationship between gender and MCS-36 in patients suffering from dizziness. The results of the current study showed no association between either the duration nor the frequency of dizziness or the severity of dizziness symptoms and the MCS-36. Given that, our results support the previous observations that clinical symptoms of dizziness seem not to be crucial for the impaired mental HRQoL [44,45] but that psychosocial factors such as emotional distress mainly contribute to the variance of the mental HRQoL [13].

Hierarchical regression of the PCS-36 explained 45 percent of its variance. The most significant associated factor was the severity of dizziness (DHI), explaining 35 percent of the variance (high DHI ~ low PCS-36). Additional four percent of the variance was explained by vertigo characteristics of dizziness, which reduced the PCS-36 further. Vestibular disturbances (e.g. spinning) and provoked autonomic symptoms such as nausea and sweating [46] potentially reduce physical activities and might influence the physical role of the patient negatively (reduced PCS-36). In addition, due to a higher severity of dizziness symptoms a vicious circle of vestibular symptoms, autonomic symptoms, and increasing physical deconditioning might set in. As a possible consequence a vicious circle of vestibular symptoms, autonomic symptoms, and increasing deconditioning might set in. In this vicious circle, adaptive processes of the central nervous system might be decelerated and dizziness symptoms might be prolonged. In turn these potentially prolonged dizziness symptoms might trigger the autonomic arousal [47,48] and possibly maintain a reduced PCS-36. As expected, high scores for sick leave were associated with low scores in PCS-36. Age and gender did not contribute significantly to the variance of PCS-36. Even though dizziness is common in older age a significant relationship between impairment in patients with dizziness and age is not obvious [49]. While univariate results suggested a significant association between HADS and PCS-36, hierarchical regression showed suppressor effects of HADS. As the univariate correlation was low the suppressor (HADS) rather improved the prediction than contributed to the variance on its own (less than one percent). Furthermore, possible suppressor effects were already indicated in the univariate context which showed a relatively high correlation between DHI and HADS and opposites signs for DHI and HADS and DHI and PCS-36.

Taken the results for PCS-36 and MCS-36 together, patients might assume a physical nature for their symptoms (low PCS-36), which might encourage them to seek medical help. If one assumes that symptoms are of somatic nature, medical visits might be unsatisfactory because no sufficient somatic cause can be found and patients might experience emotional distress by these visits. Thus, the emotional arousal potentially increases, even though the somatic impairment is unchanged or improved due to compensating processes over time [24]. This however, might result in a low MCS-36 and might explain why both the PCS-36 and the MCS-36 are reduced in patient suffering from dizziness.

Some limitations of this study should be noted. The cross-sectional design of the study prevents conclusions about causality. Longitudinal designs might reveal causal relationships between the various clinical symptoms of dizziness, psychosocial factors and HRQoL. Furthermore, the overall response rate of the study was moderate. However it is known that in non-face-to-face survey studies, it is difficult to obtain a patients response rate of 60% [50]. The non-face to-face design was used to assess the patients before the clinical examination in the Interdisciplinary Centre for Vertigo and Balance Disorders. Therefore, all our data were pre-consultation data and we do not know whether additional patient characteristics such as comorbid conditions or diagnostic subgroups might be associated with the HRQoL. However, this pre-consultation design was necessary to prevent changes in the answering behaviour due to the interdisciplinary clinical examination and counselling.

Another point is that dizziness seems to be more frequent in women [2], which is, not reflected in our sample. However, the participating patients did not differ from non-participating patients regarding gender and age. Therefore, our study seems not to suffer from a significant recruiting bias regarding these important socio-demographic variables. However, due to the pre-consultation design we were not able to compare further variables such as symptom duration, or comorbid conditions between included and excluded patients. Another limitation is the recruitment of patients at a tertiary care centre. Patients are not commonly referred to a specialist care centre with early or mild symptoms which might have resulted in patients with more severe dizziness [2,14]. This might be reflected by the somewhat higher DHI score (46.0) than in other studies [9,51]. Although we think that the current results reflect a vast array of patients suffering from dizziness, they have to be interpreted with caution.

## Conclusion

In conclusion, the current study shows that patient with dizziness suffer from significant impairments in both, the physical and the mental HRQoL. While psychosocial factors, such as emotional distress, significantly contribute to the impairment of MCS-36, clinical characteristics of dizziness mainly contribute to the impairment of PCS-36. This shows that clinical characteristics as well as the psychosocial factors have to kept in mind to improve the overall HRQoL significantly and permanently. Next to the physical examination physicians should integrate a basic psychological examination into the daily routine with dizziness patients. This could help them to identify those patients who require urgent support from interdisciplinary professional teams specialized in patients suffering from dizziness.

## Abbreviations

HRQoL: Health-related quality of life; SF-36: Medical outcomes studies 36-item short-form health survey; PCS-36: Physical component score of health-related quality of life; MCS-36: Mental component score of health-related quality of life; DHI: Dizziness handicap inventory; DHIF: Functional handicap due to dizziness (subscale of the DHI); DHIP: Physical handicap due to dizziness (subscale of the DHI); DHIE: Emotional handicap due to dizziness (subscale of the DHI); HADS: Hospital anxiety and depression scale; SD: Standard deviation; FoD: Frequency of dizziness; DoD: Duration of dizziness.

## Competing interests

The authors declare that they have no competing interests.

## Authors’ contributions

SW initiated the collaborative project, designed data collection tools, collected and monitored data collection, wrote the statistical analysis plan, cleaned, analysed, and interpreted the data, and drafted and revised the paper. ABB monitored data collection, analysed and interpreted the data, and drafted and revised the paper. DS collected data and monitored data collection, and critically revised the draft paper. SCAH collected data and monitored data collection, and critically revised the draft paper. GK collected data and revised the draft paper. MR initiated the collaborative project, monitored data collection, interpreted the data and drafted and revised the paper. All authors read and approved the final manuscript.

## Pre-publication history

The pre-publication history for this paper can be accessed here:

http://www.biomedcentral.com/1472-6963/14/317/prepub
